# A reduced lymphocyte ratio as an early marker for predicting acute pancreatitis

**DOI:** 10.1038/srep44087

**Published:** 2017-03-07

**Authors:** Xiuzhong Qi, Fangyong Yang, Haitao Huang, Yiqi Du, Yan Chen, Meitang Wang, Dezeng Zhu, Xiaoqiang Yue, Lina Wang

**Affiliations:** 1 Department of Traditional Chinese Medicine, Changhai Hospital, Second Military Medical University, No. 168 Changhai Road, Shanghai 200433, China; 2 Department of Traditional Chinese Medicine, The Qingdao First Sanitarium of Navy, No. 27 Xianggang West Road, Qingdao 266071, China; 3 Department of Intensive Care Unit, People’s Hospital, No. 1 Wenhua North Road, Laiwu, 271100, China; 4 Department of Gastroenterology, Changhai Hospital, Second Military Medical University, No. 168 Changhai Road, Shanghai 200433, China; 5 Department of emergency, Changhai Hospital, Second Military Medical University, No. 168 Changhai Road, Shanghai 200433, China; 6 Department of Traditional Chinese Medicine, Changzheng Hospital, Second Military Medical University, No. 415 Fengyang Road, Shanghai 200003, China

## Abstract

The early diagnosis and severity grading for acute pancreatitis (AP) are difficult to determine because of the complexity and differences in disease process. To date, few studies have investigated the role of lymphocyte ratio (LR) in AP. Therefore, the objective of the present study was to investigate the prognostic value of LR as an indicator in AP, as well as determine an optimal cut-off value for the severity prediction. There were two hundred four patients involved in this study, ninety-two of whom had severe acute pancreatitis (SAP). The LR was analyzed on admission and correlated with severity, which was determined using the Atlanta classification. The optimal cut-off value for LR was generated using receiving operator characteristic (ROC) curves. The results showed that the LR in the SAP group decreased significantly compared to the mild acute pancreatitis (MAP) group (8.82 vs. 13.43). The optimal cut-off value obtained from ROC curves was 0.081, with a sensitivity of 80.4%, a specificity of 53.3%, a positive likelihood ratio of 1.722, and a negative likelihood ratio of 0.368. In conclusion, the LR is obviously related to the condition of AP patients and is valuable for the differential diagnosis of SAP in early stages of AP.

Acute pancreatitis (AP) is characterized by the destruction and inflammation of pancreas tissue through the activation of pancreatic acinar cells as a result of being triggered by various factors such as biliary disease, alcoholism or hyperlipidemia^1^,^2^. It can cause significant morbidity and with a mortality rate of 5–10%^3^. The severity of AP is classified as severe acute pancreatitis (SAP), moderately severe acute pancreatitis (MSAP) and mild acute pancreatitis (MAP)^4^. The former group has a more severe illness status, more complications and higher mortality, which can reach up to 36–50%^5^. Therefore, the patients who are diagnosed with AP through clinical, laboratorial and radiological screening methods should be assessed quickly, with the goal being to select the best treatment options and improve patient outcomes. It has been stated that scoring systems such as BISAP, Balthazar CT, Ranson, and APACHE II, which usually combine symptoms and laboratory results to evaluate the patient’s condition, can be used to determine the severity of AP. However, they still need to be improved and perfected because of their complexity and low sensitivity^6^,^7^. Therefore, there is an urgent need for a fast, simple, and sensitive method to stratify patients at the time of admission or shortly thereafter.

Neutrophils, lymphocytes, endothelial cells and macrophages in the pancreatic acini are the main effector cells of AP^1^. Simple tests using serum markers, such as white cell count (WCC), hematocrit (HCT), red cell distribution width, high sensitivity C-reactive protein, procalcitonin, interleukin-6 and interleukin-8, have been shown to predict the severity of AP^8^,^9^,^10^,^11^,^12^,^13^,^14^. WCC is a routine serum hematological test that is already incorporated into many of the current AP scoring systems. However, it is easily affected by medications and other factors and easily changed under various physical and pathological conditions, which can affect its diagnostic value^15^. Researchers worldwide have suggested that we should focus on the ratios of components in the WCC, including neutrophils and lymphocytes, rather than the absolute values of white blood cells (WBCs) and their components. The absolute lymphocyte count has been assessed as an important part of the immune system and showed good prognostic value^16^. Azab has reported that the neutrophil-lymphocyte ratio (NLR) is also clearly related to a patient’s condition^15^, but turned out with controversial results^17^.

Our study is the first to compare early alterations of the lymphocyte ratio (LR) in the peripheral blood of SAP (including SAP and MSAP) and MAP patients. The purposes of this study are to determine whether the LR is a strong predictor for SAP, and how strongly it is correlated with prognosis. We also aim to determine an optimal cut-off value to differentiate between SAP and MAP patients and to provide evidence for the severity evaluation, prognosis prediction, and therapy selection for AP patients.

## Materials and Methods

### Patients

This retrospective study was approved by the institutional research and ethics committee of Shanghai Changhai Hospital, and all experiments were performed in accordance with relevant guidelines and regulations. Consecutive patients admitted to our unit with a confirmed diagnosis of AP between January 2013 and September 2015 were included. And a smaller sample of patients between November 2015 and April 2016 was collected for a further validation study, which is also retrospective. Informed consent was obtained from all participants.

AP was diagnosed by clinical findings that were consistent with a diagnosis of pancreatitis together with definitive CT manifestation^1^. Patients were classified as either SAP (including SAP and MSAP) or MAP using the Atlanta classification^4^. SAP was defined as objective evidence of organ failure (e.g., circulatory shock, acute renal failure, acute pulmonary failure) as defined in the Atlanta classification and/or local complications of necrosis (e.g., acute peripancreatic fluid collection, acute necrotic collection, walled-off necrosis, abscess of pseudocyst)^4^. Patients with MAP did not experience these complications. BISAP, modified Balthazar CT, Ranson, APACHE II and Marshall scores^6^ were calculated at baseline according to our clinical practice. The HCT, WCC, neutrophil count (NC), lymphocyte count (LC), LR and NLR were analyzed. The LR was determined by calculating the ratio of the absolute lymphocyte and total white blood cell counts.

### Statistical analysis

To compare patients with SAP and MAP, Student’s t test and logistic regression were used for continuous data, while the chi-square test and Fisher’s exact test were used for categorical data.

Receiving operator characteristic (ROC) curves were generated with corresponding area under curve (AUC) analysis and computation of 95% confidence intervals (CI) to determine the optimum LR cut-off value by showing the trade-off between sensitivity and specificity. The sensitivity, specificity, positive likelihood ratio (+LR) and negative likelihood ratio (−LR) of the cut-off value for the identification of patients with SAP were calculated. SPSS (StatisticalPackage for Social Sciences, v21) was used for the analysis. A *p* value < 0.05 indicated statistical significance.

## Results

Two hundred four patients with AP were admitted during the period of the study. Ninety-two patients had SAP (12 patients with pseudocysts) and one hundred twelve patients had MAP. There were no significant differences in age, gender, or whether the patient had received treatment prior to admission between the SAP and MAP groups. The differences in commonly used scoring systems between the SAP and MAP groups are summarized in Table 1.

Partial results of routine blood tests at admission showed that the WCC, HCT, NC and NLR of the SAP cohort had increased significantly compared to the MAP group. By contrast, the LC and the LR of the SAP cohort had decreased significantly compared to the MAP group (Table 2). The LR was finally concluded to be the most relevant factor account for multiple comparisons (Table 3), which confirmed our priori hypothesis that LR would be more useful than other factors.

**Figure 1:**
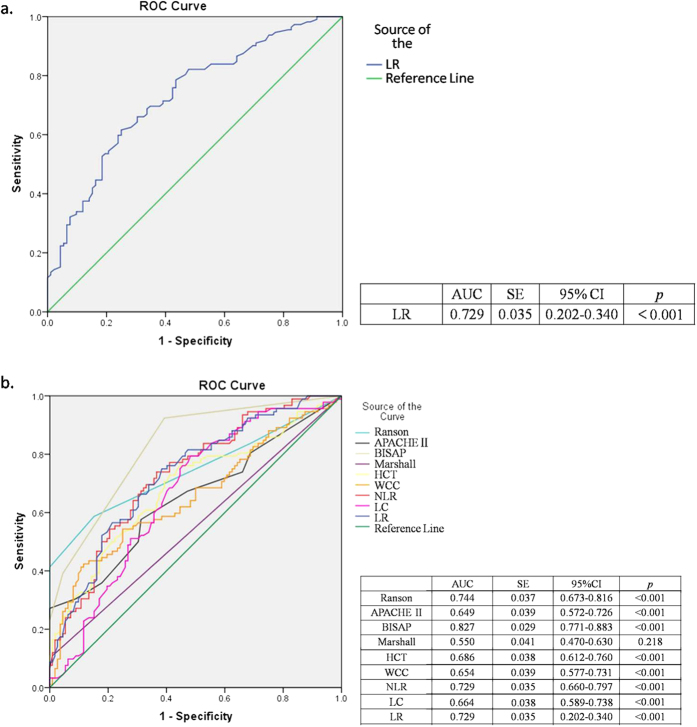
Receiver operator curves (ROC) and corresponding area under curve (AUC) analyses demonstrating the accuracy of LR as a predictor for SAP on admission (**a**), and the comparison of HCT, WCC, NLR, LC, LR and commonly used scoring systems (**b**).

The theoretical optimal cut-off value for LR and the cut-off values for diagnosis and screening were generated using ROC and corresponding AUC analyses (Fig. 1a). An optimal cut-off value of 0.081 was ultimately defined. The optimal LR was compared against other potential cut-off values by demonstrating trends in sensitivity, specificity, +LR and −LR (Supplementary Information). The verification of the validity of NLR and the comparison between the recommended cut-off value for NLR and the optimal cut-off value for LR that we defined showed that the latter had better validity as a predictor (Table 4).

A further validation study of a smaller sample was finished by analyzing another forty-four patients with a confirmed diagnosis of AP during November 2015 to April 2016. Eighteen patients had SAP and twenty-six patients had MAP. The results showed that the LR cut-off value of ≤0.081 was valuable for a differential diagnosis of SAP.

## Discussion

AP is a complex systemic inflammatory disease, and its pathogenesis is not fully understood. The early diagnosis and severity grading, as well as the treatment plan, of AP are difficult to determine because of the complicated etiology and the differences in disease process, clinical manifestation and severity. AP is associated with the development of SAP in up to 20% of patients. The mortality rate could increase to 20–50% if the best treatment opportunities are missed because of the lack of early diagnosis and severity grading^18^. In the past, only 19% of patients with AP were accurately graded, and only 67% of patients diagnosed with SAP received timely treatment in the ICU^19^. With the development of modern diagnostic technology, and particularly with improvements in scoring systems, such as BISAP, Balthazar CT, Ranson, and APACHE II, the accurate grading rate has increased to 80%^20^. Although the scoring systems have some practical clinical value, they still need to be improved and perfected because of their current complexity and low sensitivity. For example, Ranson scores, based on the results of 11 assessment factors which need at least 48 hours to get, is likely to delay the conditions of AP patients. The main disadvantages of CT scan are the high cost of the dynamic monitoring required, and the possibility of anaphylaxis of radiography agent. Marshall scores only consider the condition of SAP with functional and organic damage of the lungs and kidneys, and only involve the indexes that reflect the functions of the respiratory and urinary systems. Therefore, there is an urgent need for a simple, fast, and sensitive method to stratify patients at the time of admission or shortly thereafter.

The activation of pancreatin, the release of inflammatory mediators and the production of cytokine are considered to be primary pathogeneses of AP and involve multiple organs and systems. In AP, WBCs (including neutrophils and lymphocytes), endothelial cells, and monocyte-macrophages within the pancreas acinar are the main effector cells of the inflammatory response^1^. The WCC is part of many AP prognostic scoring systems, including the Ranson, Imrie, APACHE II, BISAP and SAPS II systems. However, the WCC is easily affected by medication and other factors and easily changed under various physical and pathological conditions, such as hydration, pressure and pregnancy. All of these can affect the absolute values of WBCs and their components^15^, making it difficult to predict the severity of AP. Therefore, scholars have suggested that we should pay attention to the ratios of components in the total WCC, including neutrophils and lymphocytes.

Lymphocyte is an important component of WBCs. It increases after initial stress and mediates the subsequent inflammatory response. Persistent lymphopenia is an independent marker of progressive inflammation, bacteremia or sepsis in emergency admissions and intensive care patients^21^. Researchers have found that the absolute lymphocyte count could be assessed as an important part of the immune system and showed good prognostic value^16^. And a recent study showed that the regulatory T cells (Tregs) were closely related to the severity of the early stage of AP, leading to the proposal of assessing Tregs as a accurate predictive factor for AP^22^. However, to date, few studies have investigated the role of LR in AP.

Based on the above studies and combined with a large number of clinical observations, our study used the LR as the research object to explore its correlation with AP severity. The LR is the percentage of LC involved in the total WCC, which is more stable under various conditions compared to the LC. The LR ranges from 20% to 40% in general circumstances. The primary finding of our study is that the LR is lower in patients presenting with AP and is able to effectively differentiate SAP and MAP patients. The change is general and there are not any specific population of lymphocytes that decreased in the severe acute pancreatitis patients. A univariate analysis showed that the WCC, NC, LC, HCT, LR and NLR differed significantly between the two groups (*P  *< 0.01). The LR was finally determined to be the most relevant factor based on a multivariable conditional logistic regression (*P  *< 0.01).

Further data analyses using ROC and corresponding AUC for the serum markers and commonly used scoring systems for AP suggest that the LR is valuable for the differential diagnosis of SAP in the early stages of AP, with a corresponding AUC of 0.729 (95% CI: 0.202–0.340). In addition, although the LR is less valuable than Ranson (AUC: 0.744, 95% CI: 0.673–0.816) and BISAP (AUC: 0.827, 95% CI: 0.771–0.883) scoring systems, it has similar clinical value for diagnosing and typing AP compared with NLR (AUC: 0.729, 95% CI: 0.202–0.340), and better clinical value compared with WCC (AUC: 0.654, 95% CI: 0.577–0.731), LC (AUC: 0.664, 95% CI: 0.589–0.738), HCT (AUC: 0.686, 95% CI: 0.612–0.760), APACHE II (AUC: 0.649, 95% CI: 0.572–0.726) and Marshall (AUC: 0.550, 95% CI: 0.470–0.630) scoring systems, which are commonly used.

By analyzing the validity of the LR as a predictor, our study determined a theoretical optimal cut-off value for the LR of ≤0.072 for identifying poor outcomes in AP. This cut-off value was extrapolated from the formula ‘D = (1 − sensitivity)^2^ + (1 − specificity)^2^ 23. The minimum of D was determine to be the theoretical optimal cut-off value, giving a sensitivity of 83.9%, a specificity of 36.9%, a +LR of 1.330, and a −LR of 0.436. However, it should be noted that the incorporation of a variable with high specificity would enhance current scoring systems, unlike avariable with high sensitivity but low specificity. Therefore, our study aims to define a cut-off value with acceptable sensitivity and greater specificity.

We considered a minimum 80% sensitivity for clinical use. This is in keeping with other scoring systems, such as the APACHE II, Imrie, Ranson, and Pancreatitis Outcome Prediction score, which report sensitivities of 60–90%^24^,^25^,^26^. Using sensitivities of 80%, 85%, and 90%, we determined cut-off values of 0.081, 0.070, and 0.063, respectively, for diagnosis, resulting in sensitivities of 80.4–90.2%, specificities of 53.3–30.4%, +LRs of 1.722–1.296, and −LRs of 0.368–0.322. The results show that higher LR cut-off values are more specific in predicting poor outcome but exhibit decreased sensitivity. By comparing the theoretical optimal cut-off value with these potential cut-off values, we finally defined an optimal cut-off value of 0.081, which has acceptable sensitivity and higher specificity.

We also observed that one of the weaknesses of the current AP scoring systems is a lack of appropriate indicators for screening. We determined cut-off values of 0.123, 0.137, and 0.151 for screening, which corresponded with specificities of 80%, 85%, and 90%, respectively, and resulted in specificities of 80.4–90.2%, sensitivities of 53.6–33.9%, +LRs of 2.735–3.459, and −LRs of 0.577–0.733. It can be conclude that lower LR cut-off values are more sensitive but less specific.

Neutrophil is also an important component of WBCs. It propagates inflammation and tissue destruction in AP by activating a cascade of inflammatory cytokines (including IL-6, IL-8 and TNF-α); proteolytic enzymes (including myeloperoxidase, elastase, collagenase and β-glucoronidase); and oxygen free radicals^27^,^28^. Studies have confirmed that the NLR, which was determined by calculating the ratio of the absolute neutrophil and lymphocyte counts, could be an independent prognostic factor for AP using a cut-off value of ≥10.6^29^. Although the NLR can predict the prognosis of AP more accurately compared with other markers^30^, it was also turned out with controversial results^17^. Our study verified the validity of the NLR using the above recommended cut-off value, and obtained a sensitivity of 80.4%, specificity of 51.1%, +LR of 1.644, and −LR of 0.384. Compared with the optimal cut-off value for the LR that we defined, the latter value has the same sensitivity, higher specificity, and better +LR and −LR, indicating that it has better predictive value for clinical use. The results also showed that the lymphocyte maybe the main effector cell of the inflammatory response in AP compared with the neutrophil.

The results of the validation study showed that the LR cut-off value of ≤0.081 was valuable for a differential diagnosis of SAP, with a sensitivity of 84.6%, specificity of 88.9%, +LR of 7.622, and −LR of 0.173.

Several limitations of our study were listed as follows: Firstly, because of the small number of the patients, the predictive power might be slightly influenced. Secondly, the retrospective nature of the study limited the extension of the study. Lastly, the collection of patients for validation study was limited by the time, which may have some influence over the results. So, in order to verify our conclusion, further larger prospective studies might be needed. The sequential changes of LR in SAP and MAP groups, and the relationship between the trends and the prognosis, also need to be observed.

## Conclusion

The LR is obviously related to the AP patient’s condition and is valuable for a differential diagnosis of SAP in the early stages of AP. An optimal LR cut-off value of ≤0.081 to identify poor outcome in AP is defined and clinical practice with small-samples has verified the validity of it. However, randomized clinical trials with large-samples are still required to furthere valuate the value of the LR in diagnosing AP at early stages, to optimize the LR, and to investigate whether its incorporation into current AP prognostic scoring systems increases the accuracy of current methods.

## Additional Information

**How to cite this article**: Qi, X. *et al*. A reduced lymphocyte ratio as an early marker for predicting acute pancreatitis. *Sci. Rep.* **7**, 44087; doi: 10.1038/srep44087 (2017).

**Publisher's note:** Springer Nature remains neutral with regard to jurisdictional claims in published maps and institutional affiliations.

## Supplementary Material

Supplementary Dataset

## Figures and Tables

**Table 1:** Patient demographics, prior treatment, and scores of commonly used scoring systems of MAP and SAP groups on admission.

	All	MAP	SAP	*p* value
Age	49.42 ± 14.76	50.51 ± 15.57	48.09 ± 13.67	0.238
Sex: male (%)	53.9	52.7	55.4	0.696
Prior treatment (%)	95.1	93.0	98.0	0.103
Commonly used scoring systems				
Ranson	1.41 ± 1.29	0.84 ± 0.67	2.11 ± 1.50	<0.001
APACHE II	3.68 ± 3.42	2.71 ± 2.41	4.87 ± 4.05	<0.001
BISAP	0.95 ± 0.98	0.44 ± 0.58	1.58 ± 1.01	<0.001
Marshall	0.11 ± 0.56	0.01 ± 0.09	0.24 ± 0.82	0.003
Balthazar CT	2.94 ± 1.08	2.02 ± 0.19	4.07 ± 0.46	<0.001

Data are expressed as the mean ± standard deviation. MAP: mild acute pancreatitis; SAP: severe acute pancreatitis.

**Table 2:** Partial results of blood routine tests comparing MAP and SAP groups on admission with a univariate analysis.

	All	MAP	SAP	*p* value
WCC (10^9^/L)	11.59 ± 4.61	10.47 ± 3.95	12.96 ± 4.99	<0.001
NC (10^9^/L)	9.62 ± 4.31	8.43 ± 3.65	11.06 ± 4.62	<0.001
LC (10^9^/L)	1.16 ± 0.46	1.27 ± 0.46	1.02 ± 0.41	<0.001
HCT (%)	38.58 ± 4.49	37.19 ± 3.54	40.28 ± 4.93	<0.001
NLR	9.80 ± 6.67	7.63 ± 4.40	12.43 ± 7.92	<0.001
LR (%)	11.35 ± 6.12	13.43 ± 6.76	8.82 ± 4.00	<0.001

Data are expressed as the mean ± standard deviation. MAP: mild acute pancreatitis; SAP: severe acute pancreatitis; WCC: white cell count; NC: neutrophil count; LC: lymphocyte count; HCT: hematocrit; NLR: neutrophil-lymphocyte ratio; LR: lymphocyte ratio.

**Table 3:** LR filtered out by multivariable conditional logistic regression.

	Variables Entered	Variables Removed				
Variables	LR	WCC	NC	LC	HCT	NLR
*p* value	<0.001	0.677	0.587	0.721	0.643	0.460

LR: lymphocyte ratio; WCC: white cell count; NC: neutrophil count; LC: lymphocyte count; HCT: hematocrit; NLR: neutrophil-lymphocyte ratio.

**Table 4:** Sensitivity, specificity, +LR and −LR of optimal cut-off value for LR defined by our study and NLR cut-off value suggested by Azab^29^.

Cut-off		Sensitivity (%)	Specificity (%)	+LR	−LR
LR	0.081	80.4 (72.9–87.8)	53.3 (42.9–63.7)	1.722	0.368
NLR	10.6	80.4 (72.9–87.8)	51.1 (40.7–61.5)	1.644	0.384

Sensitivity and specificity expressed as value (95% CI). +LR: positive likelihood ratio; −LR: negative likelihood ratio; LR: lymphocyte ratio; NLR: neutrophil-lymphocyte ratio.
