# Corrigendum: A reduced lymphocyte ratio as an early marker for predicting acute pancreatitis

**DOI:** 10.1038/srep46842

**Published:** 2017-06-07

**Authors:** Xiuzhong Qi, Fangyong Yang, Haitao Huang, Yiqi Du, Yan Chen, Meitang Wang, Dezeng Zhu, Xiaoqiang Yue, Lina Wang

Scientific Reports 7: Article number: 4408710.1038/srep44087; published online: 03 07 2017; updated: 06 07 2017

In this Article, Haitao Huang is incorrectly listed as being affiliated with ‘Department of Intensive Care Unit, People’s Hospital, No.1 Wenhua North Road, Laiwu, 271100, China’. The correct affiliation is listed below:

Laixi People’s Hospital, No. 69 Yantai Road, Laixi, 266600, China.

