# Supplementary material for: A reduced lymphocyte ratio as an early marker for predicting acute pancreatitis
Source: Sci Rep. 2017 Mar 7;7:44087. doi: 10.1038/srep44087 (PMC5339824; doi:10.1038/srep44087)
Supplement: Supplementary Dataset [file srep44087-s1.doc]

Title: **A** **reduced lymphocyte ratio as an early marker for predicting acute pancreatitis**

Author list: **Xiuzhong Qi ^1, 2, +^, Fangyong Yang ^1, +^, Haitao Huang ^3^, Yiqi Du ^4^, Yan Chen ^4^, Meitang Wang ^5^, Dezeng Zhu ^1^, Xiaoqiang Yue ^6^, Lina Wang ^1, *^**

^1^ Department of Traditional Chinese Medicine, Changhai Hospital, Second Military Medical University, No. 168 Changhai Road, Shanghai 200433, China;

^2^ Department of Traditional Chinese Medicine, The Qingdao First Sanitarium of Navy, No. 27 Xianggang West Road, Qingdao 266071, China;

^3^ Department of Intensive Care Unit, People’s Hospital, No.1 Wenhua North Road, Laiwu, 271100, China;

^4^ Department of Gastroenterology, Changhai Hospital, Second Military Medical University, No. 168 Changhai Road, Shanghai 200433, China;

^5^ Department of emergency, Changhai Hospital, Second Military Medical University, No. 168 Changhai Road, Shanghai 200433, China;

^6^ Department of Traditional Chinese Medicine, Changzheng Hospital, Second Military Medical University, No. 415 Fengyang Road, Shanghai 200003, China.

| **Schedule 1** Sensitivity, specificity, +LR and -LR of cut-off values for LR as reported by ROC analysis. | | | | | |
| --- | --- | --- | --- | --- | --- |
|  | Cut-off | Sensitivity (%) | Specificity (%) | +LR | -LR |
| [Theoretical](javascript:void(0);)optimal cut-off | 0.072 | 83.9 (77.0-90.8) | 36.9 (26.9-47.0) | 1.330 | 0.436 |
| Cut-off values for diagnosis | 0.063 | 90.2 (84.6-95.8) | 30.4 (20.9-40.0) | 1.296 | 0.322 |
|  | 0.070 | 84.8 (78.1-91.6) | 35.9 (25.9-45.9) | 1.323 | 0.423 |
|  | 0.081 | 80.4 (72.9-87.8) | 53.3 (42.9-63.7) | 1.722 | 0.368 |
| Cut-off values for screening | 0.151 | 33.9 (25.0-42.8) | 90.2 (84.0-96.4) | 3.459 | 0.733 |
|  | 0.137 | 40.2 (31.0-49.4) | 84.8 (77.3-92.3) | 2.645 | 0.705 |
|  | 0.123 | 53.6 (44.2-63.0) | 80.4 (72.2-88.7) | 2.735 | 0.577 |

Sensitivity and specificity expressed as value (95% CI)

LR: lymphocyte ratio; +LR: positive likelihood ratio; -LR: negative likelihood ratio.
